# Controlling Liquid Crystal Configuration and Phase Using Multiple Molecular Triggers

**DOI:** 10.3390/molecules27030878

**Published:** 2022-01-27

**Authors:** Linda M. Oster, Jake Shechter, Benjamin Strain, Manisha Shivrayan, Sankaran Thai Thayumanavan, Jennifer L. Ross

**Affiliations:** 1 Department of Physics, University of Massachusetts Amherst, Amherst, MA 01003, USA; osterlinda4@gmail.com (L.M.O.); jshechter@wma.us (J.S.); benjaminstrain@brandeis.edu (B.S.); 2 Department of Chemistry, University of Massachusetts Amherst, Amherst, MA 01003, USA; mmanisha@umass.edu (M.S.); thai@umass.edu (S.T.T.); 3 Department of Physics, Syracuse University, Syracuse, NY 13244, USA

**Keywords:** liquid crystals, emulsions, rod-shaped molecules

## Abstract

Liquid crystals are able to transform a local molecular interaction into a macroscopic change of state, making them a valuable “smart” material. Here, we investigate a novel polymeric amphiphile as a candidate for molecular triggering of liquid crystal droplets in aqueous background. Using microscopy equipped with crossed polarizers and optical tweezers, we find that the monomeric amphiphile is able to trigger both a fast phase change and then a subsequent transition from nematic to isotropic. We next include sodium dodecyl sulfate (SDS), a standard surfactant, with the novel amphiphilic molecules to test phase transitioning when both were present. As seen previously, we find that the activity of SDS at the surface can result in configuration changes with hysteresis. We find that the presence of the polymeric amphiphile reverses the hysteresis previously observed during such transitions. This work demonstrates a variety of phase and configuration changes of liquid crystals that can be controlled by multiple exogenous chemical triggers.

## 1. Introduction

Liquid crystals (LCs) are a material system that is responsive to stimuli such as electric and magnetic fields [1,2], changes in pH [3,4], protein binding [5,6], light [7,8,9], and temperature [10,11]. Furthermore, their optical properties are incredibly useful for displays and other read-outs of their state. Recently, liquid crystals have been used as a model system that is responsive to the molecular nature of the environment [12,13,14,15,16]. The self-organization and long-range elasticity enable liquid crystal systems to sense environmental fluctuations and react accordingly—all without a brain or a central communication network. Such responsive materials are inherently more secure than those that connect to a network, since their sensing and responding occur locally. Thus, autonomous materials of this sort that need not interact with a computer or talk to the cloud to perform a task are promising materials of the future.

The properties of liquid crystals make them outstanding material systems capable of mimicking activities performed by living organisms; exhibiting signal amplification, self-organization, and healing. Indeed, the physics of liquid crystals have also been used in models of essential biological processes such as cell division [17,18]. Inspired by biology, liquid crystals have been shown to be excellent at sensing and responding to environmental changes, making them great candidates for responsive or autonomous materials [2,19].

In order to use liquid crystals as autonomously responsive materials, we must investigate novel molecular triggers that can alter the state of the liquid crystal. Recent work has shown that surfactants can interact at air/aqueous–LC interfaces to cause transitions at the boundary, which propagate through the liquid crystal [12,14,20,21]. In this way, a local interaction results in a large-scale or macroscopic change in the state of the liquid crystal. Given the optical properties of the liquid crystal, these molecular-scale orientation changes are easily amplified and detected optically.

We have recently created designer oligomeric amphiphilic molecules that can interact with liquid crystals and alter the phase of the material [12,14]. Prior work was performed in a two-dimensional configuration, but here we examine the effects of the oligomeric amphiphiles in a three-dimensional system in the form of liquid crystal droplets made from 4-cyano-4′-pentylbiphenyl (5CB) [12,14]. We use a novel optical trapping method we previously developed to interrogate the effects of sodium dodecyl sulfate (SDS) on dynamical phase changes of the same droplet as the background solution is changed [21]. This method revealed a hysteresis in the configuration change for the same droplet when changing from bipolar to radial and back again.

**Figure 1:**
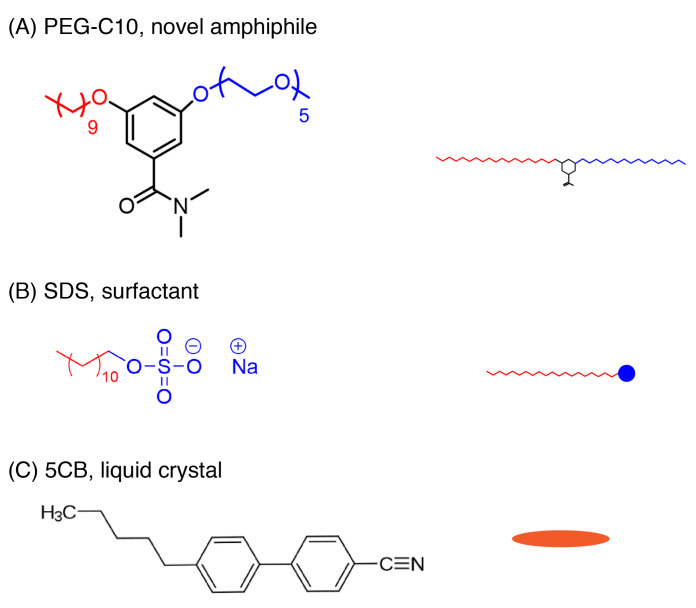
Molecular structures used in this study. (**A**) The oligomeric amphiphilic molecule, called O1 or PEG-C10. The novel functional group (shown in red), a polyethylene glycol C-10 group, is attached to the benzene ring. (**B**) Sodium dodecyl sulfate (SDS). (**C**) Liquid crystal molecule used 4-cyano-4${}^{\prime }$-pentylbiphenyl (5CB). For all images, the hydrophobic groups are shown in red and hydrophilic groups are shown in blue. The small cartoon on the right is the version used in illustrations.

Here, we characterize the activity of a new PEG-C10 surfactant, which is chemically composed of a hydrophobic decyl side chain (shown in red, Figure 1) and a hydrophilic pentaethylene glycol (PEG, shown in blue, Figure 1) side chain linked via an aromatic core. The aromatic core has a methylated amine group attached. We will refer to the oligomeric amphiphile that we examine as PEG-C10 [12,14]. We find that the PEG-C10 molecule can trigger a first nematic configuration change and then cause a melting of the liquid crystal to an isotropic phase. When we combine the PEG-C10 with sodium dodecyl sulfate (SDS), a surfactant extensively characterized for triggering liquid crystals [12,14,20,22], we alter the dynamics of the SDS activity. This work suggests that tuning the concentrations of multiple triggers can alter the driving of configuration changes in a highly controlled manner.

## 2. Materials and Methods

All chemicals were purchased from Sigma Aldrich (St. Louis, MO, USA) unless otherwise stated.

### 2.1. Synthesis of Novel Surfactant

The PEG-C10 was synthesized as described in previous work [14], where it is referred to as O1 (Oligomer 1).

### 2.2. Static Experiments

The liquid crystal droplet samples are prepared by creating a polydisperse emulsion of 5CB and surfactant in buffer. Surfactants are diluted into phosphate buffered saline (100 mM PBS, pH 7.6: 1.97 mM monobasic KH_2_PO_4_, 15.30 mM dibasic Na_2_HPO_4_, 148.86 mM NaCl). To create the emulsion, 1 μL of 5CB liquid crystal is added to the 199 μL of surfactant solution using a 10 μL Hamilton syringe (washed with chloroform and dried using compressed air). Droplets in the presence of PEG-C10 alone were observed at the following PEG-C10 concentrations: 37.5, 75, 200, 375, and 600 μM. Droplets in presence of a combination of PEG-C10 and SDS were observed with a constant PEG-C10 concentration (50 μM) and the following SDS concentrations: 100, 300, 600, 1000, 2000 μM. For all concentration of surfactants, we use standard pipettors and pipette tips that have an uncertainty of up to 20% of the lowest volume. The amounts being pipetted caused us to use pipettors with ranges from 0.2 to 2 μL all the way to 20 to 200 μL. The maximum uncertainty of our pipetting volume was 0.4 μL. Propagating that volume uncertainty to the uncertainty of the concentrations of the surfactants leads to uncertainty of our concentration from 0.04% to 2%, depending on the preparation. We do not illustrate this uncertainty on our data plots.

The LC–surfactant mixture is mechanically agitated so that the liquid crystal forms individual droplets stabilized by the surfactant. Low concentrations of surfactant serve to stabilize liquid crystal droplets and prevent coalescence, while higher surfactant concentrations are used for triggering the liquid crystal configuration change. The emulsion is pipetted into a flow chamber made from a 1 cm cloning cylinder (Fisher, Hampton, NH, USA) attached to a clean cover slip with fast-drying epoxy. The cover glass was washed with ethanol, ddH_2_O, and then ethanol to clean prior to assembly.

Samples are directly imaged using transmitted light microscopy with crossed polarizers to reveal the configuration of the nematic phase inside the droplets. Specifically, we use an inverted Nikon Ti-U microscope with a polarizer in the condenser and an analyzer in a filter cube below the objective to examine the droplets. We use a 60x 1.2 NA water immersion objective and an Andor Zyla sCMOS camera, with a pixel size of 110 nm/pixel. Nikon Elements software is used to collect data in the .nd2 file format along with its corresponding metadata. Given that we are using white light, the average diffraction limited uncertainty to measurements from the imaging is ∼250 nm. The stage is moved in the XY plane to capture droplets all around the chamber and moved in the Z direction to vary the focal plane. Movies of liquid crystal droplets in the flow chamber are recorded at 5–10 frames per second for static experiments with both surfactants (SDS and PEG-C10), and at 4 frames per minute for static experiments with just PEG-C10.

Spherical droplets were characterized based on configuration into one of the following groups: radial, bipolar, monopolar (includes destroying boojum, sunset, pre-radial, escaped radial), isotropic, or other/unknown. Additionally, the diameter of each droplet was measured and recorded.

### 2.3. Dynamic Experiments

In order to monitor the configuration of individual droplets as the environment is changed dynamically, an optical trap is used to hold droplets while the background solution is changed. A home-built optical tweezer was constructed using a fiber laser with a wavelength of 1064 nm and a maximum power of 1 W. The beam was expanded to overfill the back aperture of the objective. A three-dimensional potential well is created when the objective focuses the laser, and this holds an object of high refractive index in place at the focal plane [23,24]. We can estimate the beam waist $w_{0}$ for the laser. Using $w_{0}=\lambda /\left(\pi *NA\right)$ where λ is the wavelength of light (1064 nm) and NA is the numerical aperture of the objective (1.2), we estimate the beam waist minimum to be ∼280 nm.

**Figure A1:**
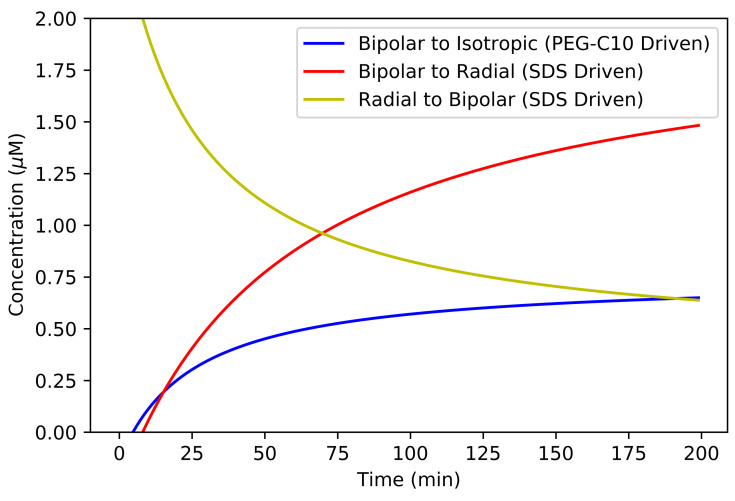
Estimated amphiphilic molecule concentration during dynamic experiments. Experiments changing from low to high SDS concentration will cause a change from bipolar to radial (red), changing from high to low SDS concentration will cause radial to bipolar transition (yellow), and changing low to high PEG-C10 concentration is the experiment being explored here (blue). These plots are found from Equation (1).

The sample is prepared using the same procedure as the static experiments. The sample at the desired starting concentration is pipetted into a flow chamber made from 1–2 stacked cloning cylinders and attached to cover glass using fast-drying epoxy. The cloning cylinders have an inner diameter of 8 mm and can hold up to 500 μL of solution. Tubing (Cole-Parmer) with an internal volume of 24.1 μL and length of 330 mm is used to flow solution into the chamber using a syringe pump (World Precision Instruments, Vernon Hills, IL, USA). Solution was flowed in at a rate of 3 μL/min. Since adding solution into the chamber caused unwanted flow, droplets trapped using optical tweezers were held opposite to the inlet tubing to reduce local flow. The concentration of surfactant (Figure A1) in the chamber can be approximated using the following equation:

(1) $$C\left(t\right)=\frac{C_{i}V_{i}+C_{f}\left(rt-24.1\right)}{V_{i}+\left(rt-24.1\right)}$$

where 24.1 represents the dead volume inside the inlet tubing (in μL), $C_{i}$ is the initial surfactant concentration, $V_{i}$ is the initial volume of solution, $C_{f}$ is the concentration of surfactant flowed in, and *r* is the flow rate (3 μL/min).

The laser power of the optical tweezer is kept in the range of 40–60%. This laser power is strong enough to hold the droplet during flow, but low enough to not trap additional droplets that flow past.

## 3. Results

### 3.1. PEG-C10 Phase Diagram

**Figure 2:**
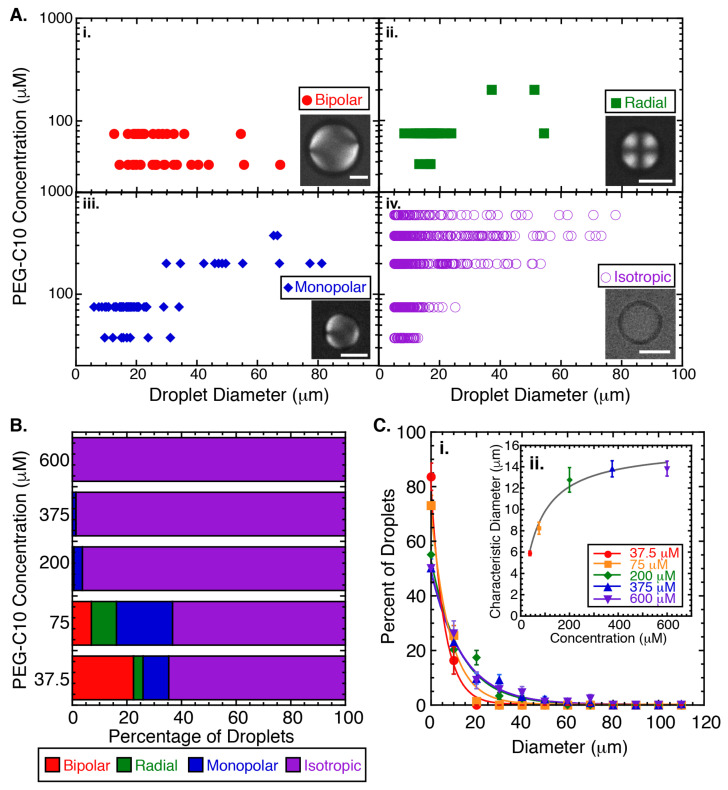
PEG-C10 controls the configuration and phase of liquid crystal droplets. (**A**) Phase diagrams of the liquid crystal configuration and diameter of 5CB droplets in presence of PEG-C10 surfactant at 37.5 μM (N = 85), 75 μM (N = 229), 200 μM (N = 355), 375 μM (N = 230) and 600 μM (N = 88). (**i**) Bipolar configuration (red circles), (**ii**) radial configuration (green squares), (**iii**) monopolar configuration (blue diamonds), and (**iv**) isotropic phase (purple open circles). Each point has uncertainty in the measurement of the width of ∼250 nm due to the diffraction limit of light, which is too small to display. (**B**) Quantification of the percentage of droplets in each configuration for each concentration of PEG-C10 for bipolar (red), radial (green), monopolar (blue), and isotropic (purple). The uncertainty of each bar is given in Appendix B.1: Table A1. (**C**) For the isotropic phase, the droplet size that is isotropic appears to increase with increasing PEG-C10 concentration. (**i**) Diameter data for each concentration (37.5 μM (red circles), 75 μM (orange squares), 200 μM (green diamonds), 375 μM (blue triangles pointing up) and 600 μM (purple triangles pointing down)). Data were binned, normalized, plotted, and fit with an exponential decay function (Appendix B.1: Table A2). Error bars represent the standard error of proportion. (**ii**) The characteristic decay time of the fit is plotted as a function of the PEG-C10 concentration and fit to a hyperbolic function (Appendix B.1: Table A3). Error bars represent the uncertainty in the fit parameters.

Our initial experiments seek to determine the phase of liquid crystal droplets in the presence of a novel oligomeric amphiphile, PEG-C10, using the following static concentrations: 37.5, 75, 200, 375, and 600 μM. Using crossed polarizers, we image the droplets and classify each as being in the bipolar configuration (Figure 2A(i)), radial configuration (Figure 2A(ii)), monopolar configuration (Figure 2A(iii)), or isotropic phase (Figure 2A(iv)). The droplets are incubated for two hours and only droplets larger than 5 μm in diameter are included in analysis.

From the phase diagram, it is clear that the isotropic phase is the favored phase for all the concentrations of PEG-C10 tested (Figure 2A,B, Appendix B.1: Table A1). Of the fraction of droplets that display the nematic phase, the configuration of the liquid crystal in the droplets follows the same pattern we have observed previously for SDS surfactant. Specifically, for nematic droplets, the initial configuration is mostly bipolar and changes to monopolar and radial. This is the expected transition for surfactants that stay at the boundary and affect the liquid crystal orientation inside the droplets [21].

The fraction of droplets that are in the isotropic phase is notably high for all PEG-C10 concentrations examined (Figure 2A,B). At the three highest concentrations, almost 100% of the droplets are in the isotropic phase, independent of the size of the droplet (Figure 2A). For the lower PEG-C10 concentrations, the smaller droplets are more likely to be isotropic than larger droplets (Figure 2A). We created normalized histograms of the diameters of isotropic droplets (Figure 2C(i)). All histograms appear to decay exponentially, likely due to the agitation method we use to the create droplets. We fit the data to an exponential decay function of the form: y(x) = A$e^{\left(-x/x_{0}\right)}$ to determine the characteristic diameter size, $x_{0}$ of the isotropic droplets (Figure 2C(i), fit data given in Appendix B.1: Table A2).

Using the characteristic size of the isotropic droplet, $x_{0}$, at each concentration, we can see that it increases monotonically with PEG-C10 concentration and appears to saturate (Figure 2C(ii)). We fit this data to a hyperbolic function of the form:

(2) $$y\left(x\right)=y_{max}\frac{x}{k+x}$$

where $y_{max}$ is the asymptote of the function at large *x*, and *k* denotes the concentration when *y* is half of $y_{max}$ (best fit information given in Appendix B.1: Table A3). Here, the maximum value denotes the characteristic diameter of the droplets due to the method of creating the droplets using agitation, since 100% of the droplets are isotropic at the highest concentrations.

The second fit parameter, *k*, denotes the concentration of PEG-C10 where the diameter of isotropic droplets is half the maximum. This concentration is likely also the concentration when half the droplets would change to isotropic, assuming that they are in equilibrium. All of the data shown here were taken at the same 2 h time point, but we cannot say for sure that we are in the equilibrium configuration. In order to examine the rate of change from nematic to isotropic, we need to quantify the configuration of the droplets over time.

### 3.2. Dynamics of LC Phase in the Presence of PEG-C10

**Figure 3:**
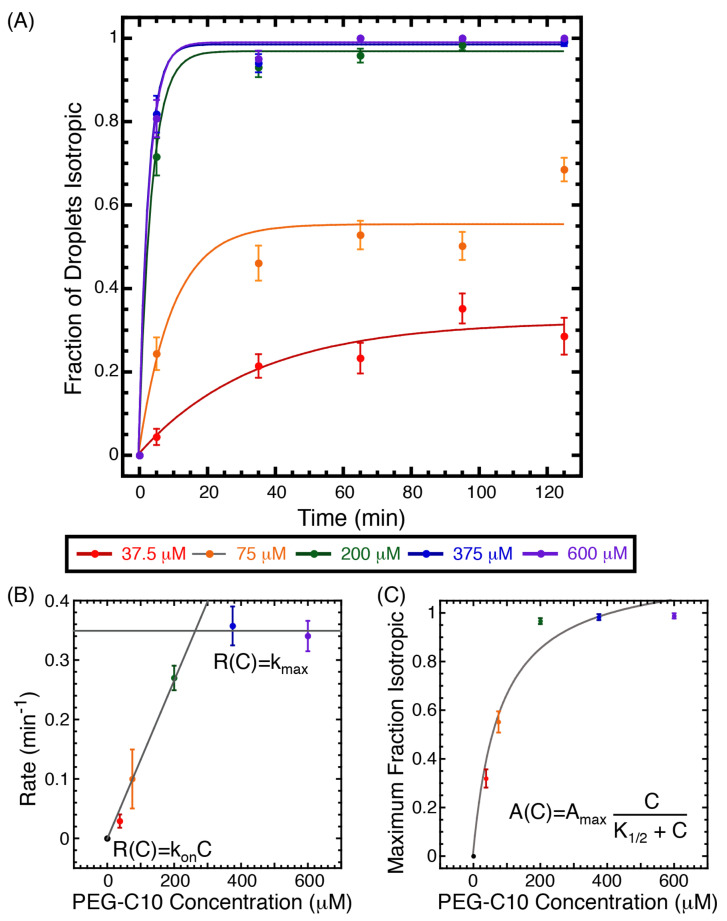
Dynamic phases over time. (**A**) The percent of droplets that are isotropic is measured over time for two hours for fixed PEG-C10 concentrations of 37.5 μM (red circles), 75 μM (orange circles), 375 μM (green circles), 600 μM (blue circles), and 750 μM (purple circles). The error bars are from the standard error of proportion using the number of droplets measured at each time point and concentration given in Appendix B.2: Table A4. Data are fit with Equation (3) represented as a solid line matching the color of the data being fit. Best fits can be found in Appendix B.2: Table A5. (**B**) The rate of change in the percentage of droplets that are isotropic is deduced from the fits in part (**A**) and plotted as a function of the PEG-C10 concentration. (**C**) The maximum percentage of droplets that are isotropic is deduced from the fits in part (**A**) and plotted as a function of the PEG-C10 concentration. Both (**B**,**C**) are fit to hyperbolic function, Equation (4). The best fit parameters are given in Appendix B.2: Table A6.

The above data are taken after two hours incubation with a fixed concentration of PEG-C10. We seek to understand if the final state is steady state and how quickly the steady-state configurations are established. In order to do this, we monitor the configuration state, focusing on the isotropic configuration, of a set of droplets over time for up to two hours (Figure 3). We test PEG-C10 concentrations of 37.5, 75, 200, 375, and 600 μM. Any droplets that did not maintain a spherical shape were discarded from the measurement. The information about the number of measurements for each time point and concentration is given in Appendix B.2: Table A4.

The percentage of isotropic droplets increased for each sample over time reaching a saturation level that was lower for the smaller PEG-C10 concentrations (37.5 and 75 μM) and plateaued at almost 100% for the higher concentrations (200, 375, and 600 μM) (Figure 3A). Each of the plots was fit to a rising exponential decay with this form:

(3) I(t)=A(1−exp(−tR)),

where I(t) is the percent of droplets that were isotropic over time, *t*; *A* is the asymptotic level of the equilibrium amount of isotropic droplets; *R* is the rate of change to isotropic. The parameters for the best fit of each fixed concentration are given in Appendix B.2: Table A5. Comparing these asymptotic maximum values from the dynamic data (Figure 3A) to the percentage of droplets that were isotropic in the equilibrium data (Figure 2B), we see the same trend, although the exact quantitative values are slightly different. That could be due to differences in preparations or times of analysis.

Using the fit to Equation (3), we can see that both the rate of change from nematic to isotropic, *R*, and the expected equilibrium value for the percentage of droplets that are isotropic, *A*, depend on the concentration of PEG-C10 (Figure 3B,C). Examining the rate of change, *R*, we note that it increases linearly for low surfactant concentrations and plateaus at higher surfactant concentrations (Figure 3B). If we fit a line to the linear portion with form: $R\left(\left[C\right]\right)=k_{on}C$, where *C* is the amphiphile concentration, and $k_{on}$ is the one-rate, we find the on-rate is best given by 0.00133 ± 0.00005 (M-min)${}^{-1}$$=2.2\times 10^{-5}$ (Ms)${}^{-1}$ (Figure 3B). Comparing this rate to the expected rate for aqueous diffusion-limited on-rates, we find it is about an order of magnitude slower than expected [25]. Most likely, this rate represents the rate of penetration of the amphiphile into the droplet interior, which results is melting the nematic to the isotropic phase.

At the two highest concentrations of PEG-C10 molecules, the transition rate, *R*, plateaus at a maximum of 0.35 min${}^{-1}$ (Figure 3B, horizontal line). This implies that at these highest concentrations, the PEG-C10 is no longer limiting the reaction; these concentrations can be considered saturating. Further, we can be confident that, at these concentrations, the data are at equilibrium, and the on and off rates (both measured in Hz) are equal to 21 Hz. This rate is also surprisingly slow, implying affinity between the PEG-C10 molecules and the 5CB.

Examining the asymptotic amplitudes for the fraction of droplets that are isotropic over time, *A*, we observe a monotonic increase with concentration (Figure 3C). Thus, the more oligomeric amphiphile present, the more droplets are isotropic at equilibrium. This corresponds to the data from the phase diagram (Figure 2B,C). These data also suggest that the two-hour time point used for steady-state measurements was acceptable as a final time point for equilibrium. The maximum *A* appears to plateau at 200 μM PEG-C10 (Figure 3C), where almost 100% of the droplets are in the isotropic phase at equilibrium. Aqueous reactions depend on the concentration of the reactants hyperbolically with the form:

(4) $$A\left(C\right)=A_{max}\frac{C}{K_{1/2}+C},$$

where A(C) is the maximum fraction of droplets that are isotropic as a function of PEG-C10, *C*, at long times (the asymptote of the data in (Figure 2A)), $A_{max}$ is the maximum amplitude plateau, which is constrained to be 100% by the experiment, but is a free-floating parameter of the fit, and $K_{1/2}$ which is the concentration where 50% of the droplets are isotropic. When the $I_{max}$ is able to be a fit parameter, the best-fit value was 119% ± 9%. In this case, the best-fit value of $K_{1/2}$ is 80 ± 20 μM. If we fixed the $I_{max}$ to 100%, the $K_{1/2}$ value becomes 50 ± 10 μM, which is a more reasonable value, given the data. Comparing these two different fits, the $\chi^{2}$ value is lower when the $I_{max}$ is higher (see fit parameters in Appendix B.2: Table A6). The characteristic concentration for the transformation from nematic to isotropic, $K_{1/2}∼$ 50–80 μM PEG-C10, should be the most sensitive for controlling the transition.

### 3.3. Dynamic PEG-C10 Effects on Individual Liquid Crystal Droplets

The data show that the fraction of droplets that are isotropic increases with concentration, that the smallest droplets are more likely to be isotropic (Figure 2), and the the fraction of droplets that are isotropic increases with time (Figure 3), all suggest that the PEG-C10 amphiphile is causing the transition from nematic to isotropic by entering the droplet from the aqueous phase into the liquid crystal. In order to directly observe the transition for a single droplet, we use an optical trap to hold a liquid crystal droplet while the background fluid is changed. This allows us to directly observe any configurational or phase changes that might occur due to the addition of the PEG-C10. The concentration of the PEG-C10 was changed from 75 to 750 μM and only larger droplets were used (>20 μm in diameter).

**Figure 4:**
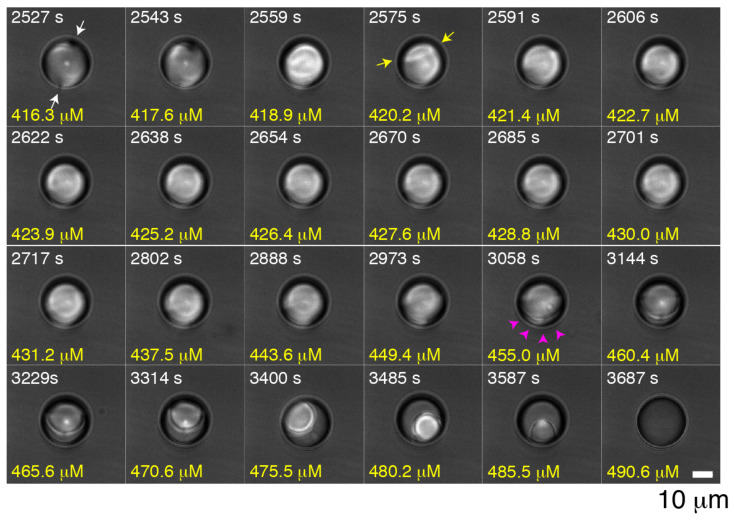
Example time series of images of an optically trapped liquid crystal droplet in the presence of changing PEG-C10 concentrations (bottom yellow numbers) over time (white top numbers). Initially, the droplet is in a bipolar configuration (*t* = 2527–2543). White arrows denote the location of the two poles. The droplet changed configuration and begins to merge its poles through the formation of a ring defect, denoted with yellow arrows at time 2575 s. The ring defect is visible for several frames, but does not transition to a radial droplet. Instead, the edge of the droplet begins to transition, making the nematic phase smaller and smaller inside the droplet. At time 3058 s, the edge of the nematic phase is seen moving inwards as it is overpowered (pink arrowheads). The nematic phase shrinks and the droplet is free to rotate in the optical trap, rotating the nematic region. At time 3687 s, the droplet has completely transformed into a isotropic phase. Scale bar is 10 μm for all frames.

Each droplet begins in the bipolar configuration with two poles on opposite sides (i.e., Figure 4, 2527 s, 416.3 μM, white arrows). This is the configuration expected with little surfactant on the surface—just enough to keep the droplets from coalescing. As the concentration increases, one of the two bipolar defects becomes a traveling ring defect (Figure 4, 2575 s, 420.2 μM, yellow arrows). In our prior work, a traveling ring defect was predicted from simulations of liquid crystals in droplets. We used this optical trapping technique to directly observe the traveling ring defect when droplets transitioned from bipolar to radial configurations and compared this to theoretically predicted intermediate configurations [21].

Unlike our prior results with SDS where a radial configuration followed the ring defect motion [21], here, in the presence of PEG-C10, the nematic phase stays in an intermediate state. Interestingly, radial droplets are observed in the steady-state configuration diagram (Figure 2A,B) at both 37.5 and 75 μM PEG-C10 and mostly observed in droplets with diameters between 5 and 20 μm. We do not see the radial configuration at higher concentrations. Since we use larger droplets for optical trapping experiments, we start the concentration of PEG-C10 at 75 μM, and we require the droplet to be in the bipolar configuration to start, it is not surprising that we do not see the radial configuration. Indeed, these dynamics data imply that the liquid crystal might need to be a smaller volume for the 75 μM PEG-C10 to enable the radial configuration.

Instead of becoming radial, the droplet stays in an intermediate configuration until the nematic phase begins to melt, becoming an isotropic phase (Figure 4, 3058 s, 455.0 μM, pink arrows). We directly observe the front of the phase transition from nematic liquid crystals to isotropic—this is the boundary between the ordered and melted states. The front moves from the edges of the droplet to the center until the droplet is completely melted. Because droplets are free to rotate even when held by optical tweezers, the spherical nematic region appears to change position throughout time series (i.e., Figure 4, 3144–3587 s frames). We cannot control the rotation of the droplet using the optical trap system here, but if the polarization of the incident beam were modified, the location, rotation, and speed of the rotation could be modified in future studies. In the current study, the location of the nematic region within the droplet is purely incidental, although we notice that the nematic phase often stays to one side so that it is in contact with the aqueous phase of the emulsion. This may suggest that the nematic phase has a higher surface affinity for the aqueous interface over the isotropic portion of the droplet.

**Figure 5:**
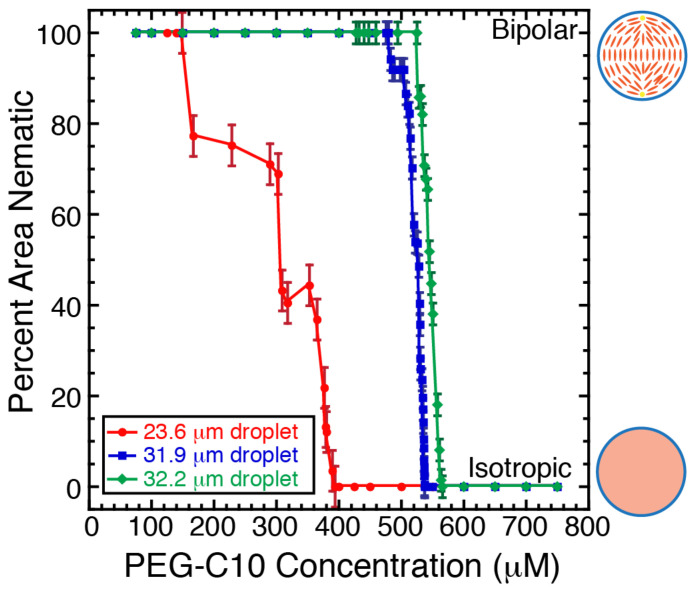
Projected area that was isotropic as a function of background concentration of monomer during dynamic PEG-C10 exchange in optically trapped droplets. Three droplets shown are 23.6 μm in diameter (red circles), 31.9 μm in diameter (blue squares), and 32.2 μm in diameter (green diamonds). When the droplets are in the bipolar state or traveling ring state, they are 100% nematic. When the droplets are completely isotropic, they are 0% nematic. Error bars represent cumulative uncertainty from measuring distances from diffraction-limited images.

Using the images of the droplets in the optical trap, we measure the projected size of the nematic region over time. Specifically, we measure the diameter and calculate a projected nematic area (μm${}^{2}$). We normalize the nematic area measurement by dividing by the total projected area of the droplet. Since we know the relationship between the time in the movie and the concentration of PEG-C10 added (Equation (1)), we can plot the fraction of droplet that is in the nematic phase as a function of the PEG-C10 concentration (Figure 5). For two larger droplets (∼32 μm diameter), the initial PEG-C10 of transformation and the rate of transformation are similar, and the rate of melting transition appears fairly constant. It appears that the smaller droplet transitions before the larger ones with a less consistent rate of melting. As the radius of the droplet decreases, the surface area to volume ratio increases, so it is expected that the smaller droplet should transform before larger droplets. The same trend was observed in the phase diagrams that showed that smaller droplets at low PEG-C10 concentrations were more likely to be isotropic than larger droplets (Figure 2C).

The concentrations of the transition we measure for the trapped droplets are far higher than the $K_{1/2}$ concentrations needed to cause the transition in half the droplets. We would expect such large droplets to take longer to change, but even a lower concentration of PEG-C10, such as 200 μM should be able to cause the change (Figure 2 and Figure 3). Indeed, that is the concentration that triggers the smaller droplet to change (Figure 5, red). For the larger droplets, by the time the melting is visible, the concentration is already saturating, as seen from the dynamic experiments (Figure 3). Thus, the transition appears to occur fast, but in reality it was already delayed compared to expectations from equilibrium measurements. That delay could be due to the time to shift the concentration in the experiment or a lag in the transition due to the droplets being so large and amount of PEG-C10 needed to penetrate the droplet to cause the change.

### 3.4. Configuration of Droplets in the Presence of SDS and PEG-C10

Our results above show that the addition of our oligomeric amphiphile PEG-C10 molecules results in two distinct transitions of the liquid crystal configuration. First, there is a rapid surface-induced transition. That resulted in the bipolar configuration changing to the monopolar configuration. After that, the surfactant acts to disrupt ordering in the droplet by penetrating the droplet in a diffusion-limited manner. In our dynamic studies using the optical trap, we were not able to reverse the state from isotropic back to nematic through dilution. Instead, we propose that a second surfactant could be applied to alter the state of the droplets. We have previously successfully worked with SDS as a surfactant to trigger droplet configuration changes [21]. Here, we mix SDS with the PEG-C10 to determine if the SDS can reverse or overpower the phase change induced by the PEG-C10.

**Figure 6:**
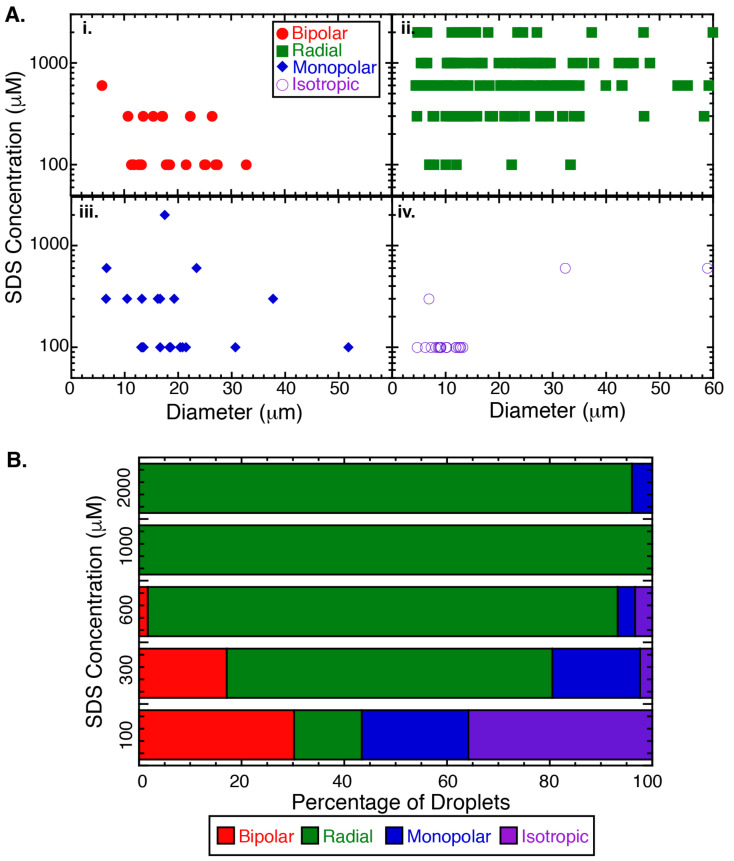
SDS and PEG-C10 control the configuration of liquid crystal droplets. (**A**) Phase diagrams of the liquid crystal configuration and diameter of 5CB droplets in presence of 50 μM PEG-C10 and SDS at the following concentrations: 100 μM (N = 45), 300 μM (N = 43), 600 μM (N = 61), 1 mM (N = 40), and 2 mM (N = 25). Configurations identified include (**i**) Bipolar (red filled circles), (**ii**) Radial (green filled squares), (**iii**) Monopolar (blue, filled diamonds), and (**iv**) isotropic (open purple circles). (**B**) Quantifying the percentage of droplets at each concentration of SDS that display the bipolar (red), radial (green), monopolar (blue), or isotropic (purple) configurations.

We first characterize the steady-state morphology of droplets at fixed SDS and PEG-C10 concentrations, 50 μM, near measured $K_{1/2}$. We alter the SDS concentrations in solution: 100 μM, 300 μM, 600 μM, 1 mM, and 2 mM. These concentrations are chosen to be well below the critical aggregate concentration (CAC) of SDS, 10 mM (Figure 6). At the lowest SDS concentration, 100 μM, the majority of droplets are isotropic (36±7%) or bipolar (30±6%), implying that this low concentration of SDS could not fully inhibit the effects of the PEG-C10 (Figure 6). Increasing the SDS concentration to 300 μM decreases the incidence of isotropic droplets to only 2±2%. The majority of droplets at 300 μM are in the radial configuration (63±8). At 600 and 1000 μM SDS, nearly all droplets are radial (92±4% and 100% respectively) and no isotropic droplets were found at 1 and 2 mM SDS.

### 3.5. SDS-Driven Dynamic Transitions in the Presence of PEG-C10

**Figure 7:**
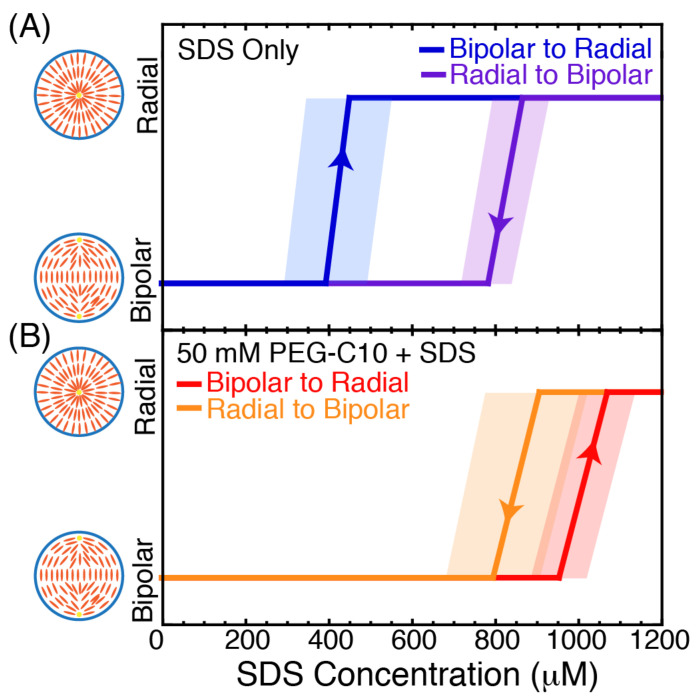
Imaging of optically trapped 5CB droplets during dynamically changing the SDS concentration. (**A**) Mean transition concentrations for bipolar to radial (blue, N = 7) when the SDS concentration is increased and radial to bipolar (purple, N = 8) when the SDS concentration is decreased. As previously reported, there is a hysteresis in the transition depending on the direction [21]. Shaded regions represent the standard error of the mean for the transition concentrations. (**B**) Mean transition concentrations in the presence of 50 μM PEG-C10 for bipolar to radial (red, N = 11) when the SDS concentration is increased and radial to bipolar (orange, N = 8) when the SDS concentration is decreased. Shaded regions represent the standard error of the mean for the transition concentrations.

As above, we use an optical trap to hold a single droplet in place and change the background surfactant concentration to directly observe the configuration. We previously performed these experiments in the presence of SDS [21], and found consistent concentrations for transition from bipolar to radial and radial to bipolar, which we repeat here (Figure 7A). As previously shown, the concentration for the configuration change is not the same, and there is a hysteresis where the SDS is able to trigger a change to bipolar at lower concentrations when being added compared to the concentration when it is being removed.

When we repeat the same experiment in the presence of 50 μM PEG-C10, we see a very different response. Both the transition concentrations are increased compared to SDS alone, occurring at 1000 ± 70 μM for the transition from bipolar to radial and 900 ± 100 μM for the transition from radial to bipolar (Figure 7B). The other noticeable difference is that the transition concentrations are reversed compared to the SDS only case. Specifically, more SDS must be added to become radial and more must be removed to revert back to bipolar. Thus, there is still hysteresis in the configuration change, but the difference is smaller and the opposite sign compared to SDS alone. We conjecture that this difference may be due to the PEG-C10 entering the droplet and making the transition harder for the surface SDS to trigger. Future modeling work may reveal that the existence of the PEG-C10 in the interior of the droplet, which presumably prefers the isotropic phase, is fighting the SDS for orientation of the liquid crystal. Increasing the PEG-C10 would likely overwhelm the SDS and cause the droplet to become isotropic.

## 4. Discussion

Using molecules to drive the configurational change of liquid crystals is a model system for molecular-to-macroscopic triggering which is useful in automatic materials systems. Here, we use a novel oligomeric amphiphile in a microscale three-dimensional geometry of 5CB liquid droplets.

It appears that the PEG-C10 designer amphiphile is able to cause two transformations of the liquid crystals in droplets. The first is a transition in configuration of the nematic from bipolar to monopolar or radial. In the static experiments, we observed radial and monopolar droplets, but in the optical trap experiments, we only saw monopolar droplets with the ring defect. After that first transition, the amphiphile is able to invade the interior to cause the nematic phase to transition to an isotropic one. This transition comes from the penetration of the amphiphile molecules into the interior of the droplet from the surface. If there is enough surfactant and enough time, the entire droplet will become isotropic.

A similar result was observed in a two-dimensional geometry with similar designer molecules [14]. In those prior works, 5CB molecules were coated onto electron microscopy grids and submerged in aqueous solution. The nematic phase shifted anchoring to change the configuration of the liquid crystal at short times in the presence of oligomeric surfactants. Over a long time, all nematic ordering was destroyed. In that system, the transitions took over 30 h. One benefit of the liquid crystal droplets is the fast dynamics for these transitions.

**Figure 8:**
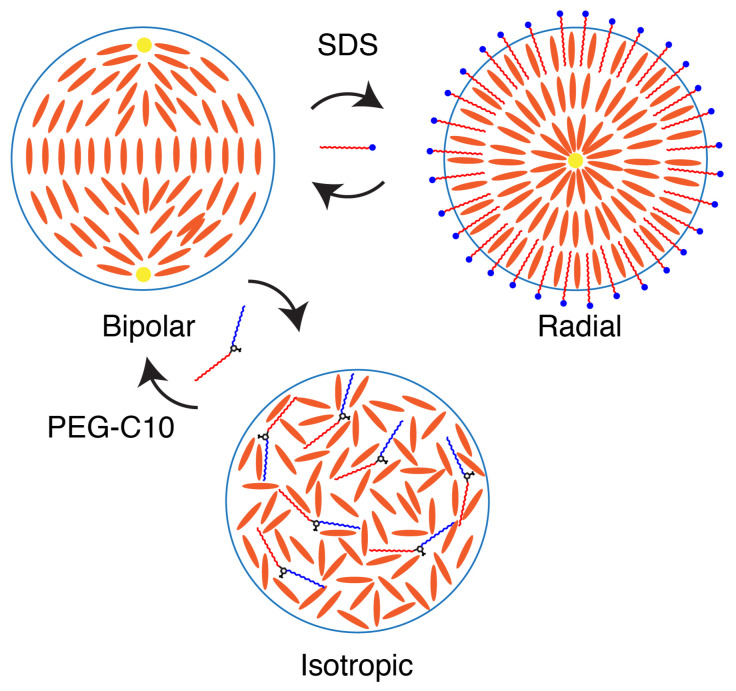
Cartoon depicting the configurations and states of 5CB (blue ovals) droplets. In a low concentration of surfactant, the droplets are stabilized to be separate and are bipolar. The addition of SDS causes a configurational change from bipolar to to radial because the SDS molecules bind at the interface. This change is reversible when the SDS concentration is lowered. In the presence of the PEG-C10, the nematic phase is disrupted and becomes isotropic because the amphiphile enters the droplet.

For larger droplets in which we could directly observe the phase transition, we saw the configuration change from bipolar to monopolar after the ring transition. The nematic ordering then began to be annihilated as the amphiphile traversed the boundary into the droplet’s interior (Figure 8). Using the dynamics data, we found that the rate of change from nematic to isotropic was not a snap-transition, like the configuration changes. Rather, it was slower and diffusion limited. In our system, the droplets are smaller and have a larger surface area to volume ratio compared to prior two-dimensional configurations [14]. Thus, the transition from ordered to isotropic was completed much faster; in several hours rather than a day or more.

**Figure 9:**
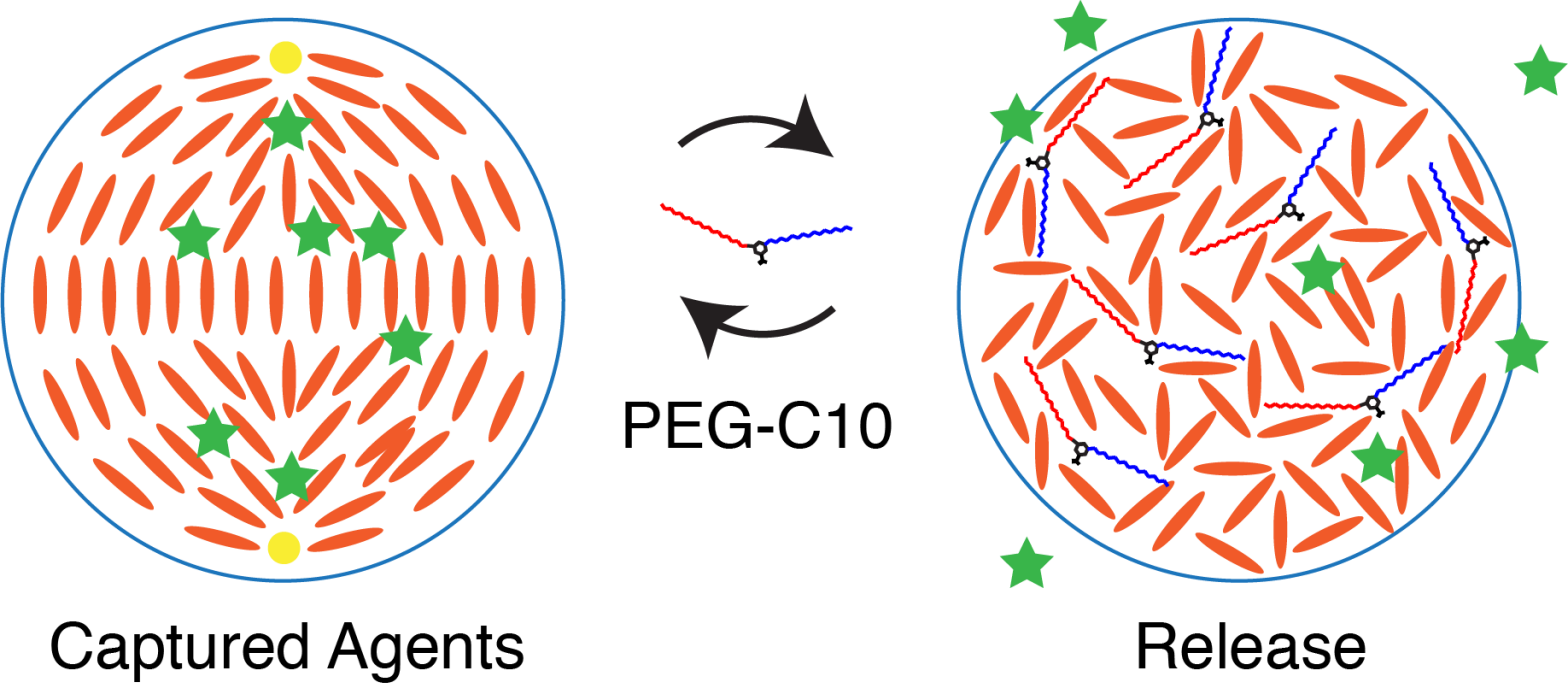
In this cartoon, cargo (green stars) are contained and trapped within the liquid crystal droplet due to the induced bipolar configuration. Upon increasing the concentration of monomer amphiphile, liquid crystal organization is destroyed, allowing for the release of cargo. This system could be fine tuned to allow for timed cargo release. Cartoon not to scale.

One exciting effect of purposefully destroying the nematic state in liquid crystal droplets could be to expose or release cargo molecules housed in the liquid crystal. In this possible scheme, a cargo molecule could be trapped by the nematic phase of the liquid crystal. When the amphiphile causes a nematic to isotropic phase transition, these carrier molecules could be released (Figure 9).

Although the amphiphile molecule was able to produce a transition from nematic ordering to isotropic, the isotropic state in liquid crystals is difficult to read optically or electronically. Thus, we tested if a previously used surfactant, SDS, was capable of overcoming the disorder caused by the amphiphile. Indeed, we found that the SDS, at high enough concentration, is able to maintain the nematic state and alter that state, even in the presence of the novel amphiphile. Multiple triggers of various molecules could cause driving into different configurations and phases as needed (Figure 8).

Interestingly, the hydrophobic regions of the designer amphiphile molecule are not very different from that of SDS, but the hydrophilic regions are very different. Specifically, the polymeric nature of the novel amphiphile may be what allows it to enter into the droplet and disrupt the nematic phase. If the hydrophilic part is able to self-associate in some way, such as fold into itself like an amino acid would, this could not only reduce the energy barrier to entering, but also could be bulky enough to disrupt the droplet’s nematic ordering (Figure 8).

Next steps for this study could include fluorescently labeling the PEG-C10 and SDS surfactants and observing the samples under the conditions presented in this work to see if the difference in behavior is indeed due to the PEG-C10 crossing the aqueous–LC interface or whether this disruption occurs by some other mechanism. Further, molecular dynamics models of the designer amphiphiles could determine if the molecules are able to fold to enable entering the liquid crystal phase and disrupt the order.

## 5. Conclusions

Liquid crystals are important chemicals for industrial and medical applications. Their ability to respond and report out on the environment has made them useful in myriads of applications already and an obvious target of study for responsive materials systems of the future. Here, this study examines the effect of a novel amphiphilic molecule on the phase of liquid crystal within a micron-scale droplet in a 3D configuration. Emulsions of liquid crystal droplets are novel composite materials with phase separated aqueous and organic phases that allow separation of materials and multi-responsive functionality. We show that the novel amphiphile is able to cause configuration changes and disrupt nematic ordering in 5CB liquid crystal droplets depending on concentration of surfactant and duration of time. Further, we demonstrate that the addition of a well-characterized surfactant, SDS, is able to overcome the isotropic transition at lower amphiphile concentration, thereby restoring nematic ordering. We performed these experiments using an optical tweezer to trap and characterize individual droplets given more information than ever before on the mechanisms of the transformations in state and phased. Future studies using the optical trap could work to not only characterize, but also manipulate individual droplets by tuning the laser power and polarization while holding the droplets.

## Data Availability

All supporting data are available upon request from the authors.

## Abbreviations

The following abbreviations are used in this manuscript:

LC	Liquid crystal
SDS	Sodium dodecyl sulfate
Novel amphiphile	PEG-C10

## Appendix B. Appendix Tables

### Appendix B.1

**Table A1:** Data and uncertainty for percentage data given in Figure 2B. Uncertainty calculated as the standard error of proportion.

Concentration μM	% Bipolar	% Radial	% Monopolar	% Isotropic	N
37.5	22 ± 5	4 ± 2	9 ± 3	65 ± 5	85
75	7 ± 1	9 ± 2	20 ± 3	63 ± 3	229
200	0 ± 0	0.6 ± 0.3	3 ± 1	96 ± 1	355
375	0 ± 0	0 ± 0	1.3 ± 0.7	99 ± 1	230
600	0 ± 0	0 ± 0	0 ± 0	100 ± 0	88

**Table A2:** Exponential fits to the normalized histograms of the droplet diameters for each concentration shown in Figure 2C(i). The fit equation used is: $y\left(x\right)=A\exp-x/x_{0}$.

Concentration (μM)	Amplitude, *A*	Characteristic Diameter, $x_{0}$	$\chi^{2}$
37.5	0.84 ± 0.01	5.9 ± 0.2	0.0000922
75	0.74 ± 0.02	8.2 ± 0.6	0.00438
200	0.54 ± 0.02	13 ± 1	0.00613
375	0.50 ± 0.01	13.8 ± 0.8	0.00204
600	0.50 ± 0.01	13.8 ± 0.7	0.00179

**Table A3:** Hyperbolic function fits to the characteristic droplet diameters for each concentration shown in Figure 2C(ii). Fit equation is (2).

Amplitude, $Y_{max}$	Half-Maximum Concentration, *k*	$\chi^{2}$
15.9 ± 0.6	63 ± 9	0.981

### Appendix B.2

**Table A4:** N-values for each time and concentration point used in Figure 3A.

Time (min)	37.5 μM	75 μM	200 μM	375 μM	600 μM
5	113	119	102	77	78
35	210	141	115	117	122
65	133	212	145	71	101
95	176	223	120	130	135
125	105	267	151	108	122

**Table A5:** Best fits for data in Figure 3A.

PEG-C10 Concentration (μM)	*A*	*r* (s${}^{-1}$)	$\chi^{2}$
37.5	32 ± 4	0.03 ± 0.01	50.0
75	55 ± 4	0.10 ± 0.05	270
200	97 ± 1	0.27 ± 0.02	23.6
375	98 ± 1	0.36 ± 0.03	24.7
600	94 ± 1	0.34 ± 0.03	18.2

For Equation (4), there are two options. We can allow $I_{max}$ to be a fit parameter (unconstrained), or we can fix it at 100% (constrained). The fit parameters are given here, with the $\chi^{2}$ value for each for comparison.

**Table A6:** Comparison of fits for data in Figure 3C.

Fit Type	$I_{max}$	$K_{1/2}$	$\chi^{2}$
Unconstrained	119 ± 9%	80 ± 20	217
Constrained	100%	50 ± 10	562

### Appendix B.3

**Table A7:** Data and uncertainty for percentage data given in Figure 6B. Uncertainty calculated as standard error of proportion.

Concentration μM	% Bipolar	% Radial	% Monopolar	% Isotropic	N
100	30 ± 6	13 ± 5	21 ± 6	36 ± 7	53
300	17 ± 6	63 ± 8	17 ± 6	2 ± 2	41
600	2 ± 2	92 ± 4	3 ± 2	3 ± 2	59
1000	0 ± 0	100 ± 0	0 ± 0	0 ± 0	40
2000	0 ± 0	96 ± 4	4 ± 4	0 ± 0	25
